# Alkoxy‐Substituted Anthrabis(Thiadiazole)‐Terthiophene Copolymers for Organic Photovoltaics: A Unique Wavy Backbone Enhances Aggregation, Molecular Order, and Device Efficiency

**DOI:** 10.1002/asia.202500678

**Published:** 2025-06-23

**Authors:** Yi Yan, Hiroki Mori, Tomoki Yoshino, Ryuki Inami, Jiaxin Chang, Junqing Gao, Yasushi Nishihara

**Affiliations:** ^1^ Research Institute for Interdisciplinary Science Okayama University 3‐1‐1 Tsushimanaka, Kita‐ku Okayama 700–8530 Japan; ^2^ Graduate School of Environmental, Life, Natural Science and Technology Okayama University 3‐1‐1 Tsushimanaka, Kita‐ku Okayama 700–8530 Japan

**Keywords:** Aggregation, Backbone conformation, Conjugated polymers, Organic solar cells, Semiconducting polymers

## Abstract

Two polymer donors, **PATz3T‐o6BO** and **PATz3T‐o6HD**, incorporating alkoxy‐substituted anthra[1,2‐*c*:5,6‐*c*′]bis([1,2,5]thiadiazole), were strategically designed and synthesized. The unique wavy backbone of these polymers effectively reduced aggregation, leading to enhanced solubility and significantly improved molecular ordering. Consequently, the **PATz3T‐o6HD**:**Y12**‐based solar cells achieved a power conversion efficiency (PCE) of 7.94%. These findings provide valuable insights into the molecular design of high‐performance polymer donors for organic photovoltaics (OPVs).

## Graphical abstract

By incorporating a lower‐symmetry terthiophene donor unit in place of quaterthiophene, the resulting ATz‐based polymer donors exhibit reduced aggregation, improved solubility, and a favorable shift in molecular orientation from edge‐on to face‐on. As a result, solar cells based on the **PATz3T‐o6HD**:**Y12** system demonstrated a remarkable improvement in PCE, increasing from 0.9% to 7.9%.

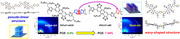

## 1 Introduction

**Figure 1:**
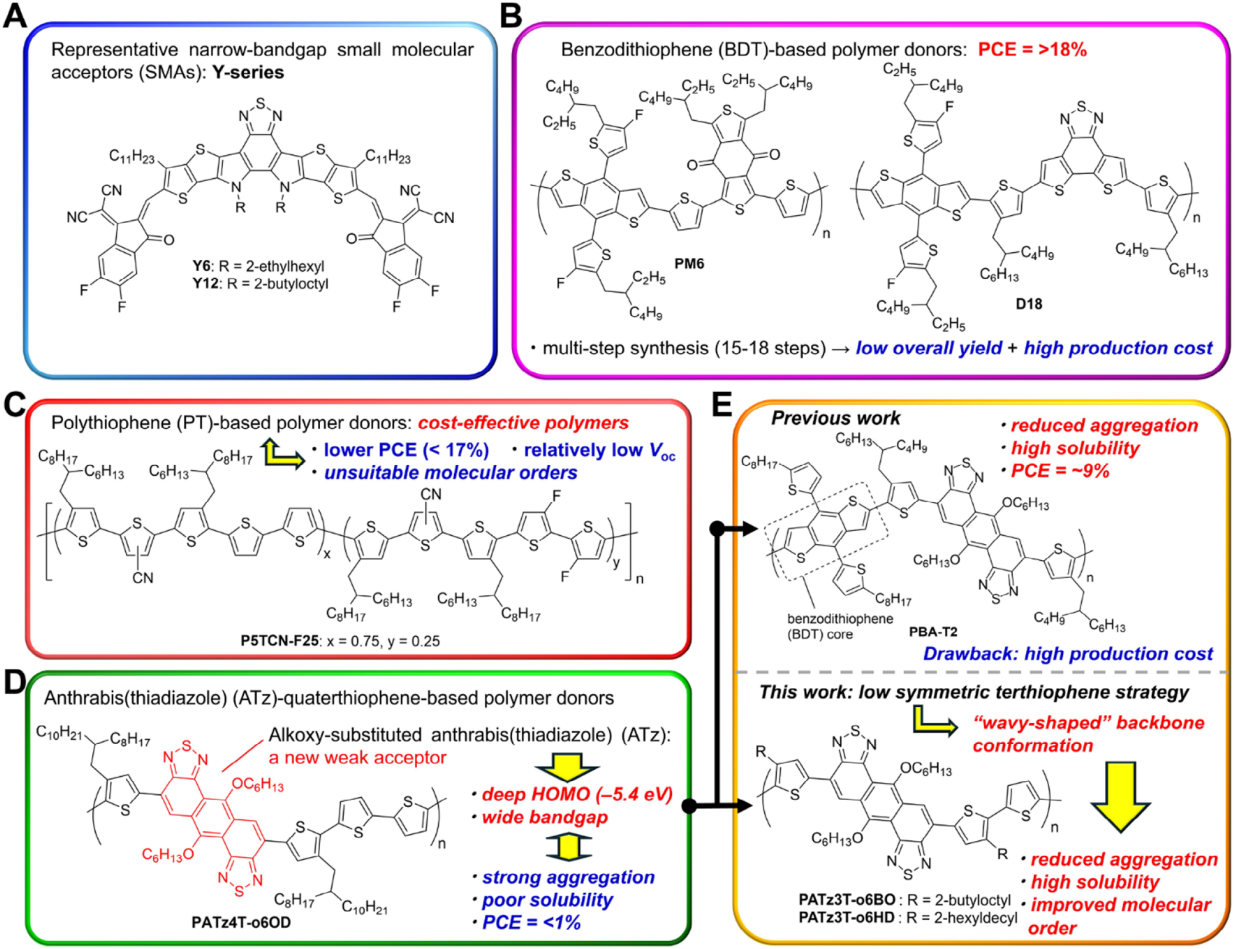
Structures of **Y6**, **Y12**, **PM6, D18**, **PATz4T‐o6OD**, **PBA‐T2**, **PATz3T‐o6BO**, and **PATz3T‐o6HD**.

Nonfullerene organic photovoltaic cells (NF‐OPVs) composed of wide‐bandgap polymer donors and narrow‐bandgap small molecular acceptors (SMAs) have seen remarkable advancements in recent years.^[^ ^1^ ^]^ In particular, the rapid development of various SMAs, such as the **Y6** and **Y12** series (Figure 1A), has significantly boosted the power conversion efficiencies (PCEs) of NF‐OPVs beyond 20% at the laboratory scale.^[^ ^2^, ^3^, ^4^, ^5^, ^6^ ^]^ However, despite these advances, reports on high‐performance polymer donors remain relatively scarce compared to those on SMAs. Consequently, the development of novel wide‐bandgap polymer donors is of critical importance. Among the polymer donors developed to date,^[^ ^7^, ^8^, ^9^, ^10^ ^]^ **PM6** ^[^ ^2^, ^11^ ^]^ and **D18** ^[^ ^3^, ^5^, ^6^, ^12^ ^]^ have emerged as the most versatile due to their exceptional PCEs exceeding 18% (Figure 1B). However, a major drawback of both polymers is their complex multistep synthesis (15–18 steps), which leads to low overall yields and high production costs.^[^ ^11^, ^12^ ^]^ Therefore, the rapid development of high‐performance and cost‐effective polymer donors is essential for the practical and industrial implementation of NF‐OPVs.

Polythiophene (PT) and its derivatives are among the most promising candidates for cost‐effective, high‐performance polymer donors in OPV development due to their simple molecular structure, ease of synthesis, and low‐cost raw materials.^[^ ^13^ ^]^ For instance, solar cells incorporating **P5TCN‐F25** (Figure 1C) and **Y6** have demonstrated an impressive PCE of 16.6%.^[^ ^14^ ^]^ However, the PT‐based polymer donor **P5TCN‐F25** is unsuitable for large‐area production due to its linear polymer backbone, which predominantly adopts an edge‐on orientation and performs poorly in thick films (>300 nm).^[^ ^14^ ^]^ In addition, OPVs based on these polymer donors often exhibited relatively low *V*_oc_ in the range of 0.7–0.8 V, which can be attributed to their high‐lying HOMO energy levels.^[^ ^13^, ^14^ ^]^ Therefore, the development of polythiophene‐based polymer donors incorporating weak acceptor units is critical for the successful industrialization of OPV technologies.

To control frontier energy levels, backbone conformation, and molecular ordering, the design of donor–acceptor (D–A) polymers incorporating a π‐extended electron‐accepting unit alongside a polythiophene‐like donor unit is a commonly employed strategy. For instance, anthra[1,2‐*c*:5,6‐*c*′]bis([1,2,5]thiadiazole) (ATz) is a thiadiazole‐containing acceptor unit with tunable 6,12‐functionalization. It features a rigid anthracene core fused to two thiadiazole rings, imparting strong electron affinity, low‐lying HOMO/LUMO energy levels, and enhanced π‐orbital overlap. These characteristics facilitate proper π‐stacking and long‐range molecular ordering in the resulting polymers.^[^ ^15^, ^16^, ^17^ ^]^ In our previous work, we developed the D–A polymer **PATz4T‐o6OD** by integrating an alkoxy‐substituted ATz derivative with quaterthiophene (Figure 1D).^[^ ^15^ ^]^ Although **PATz4T‐o6OD** possesses a rigid, π‐extended structure, its strong tendency to aggregate severely limits its solubility. When blended with **Y6**, the resulting films were cracked and inhomogeneous, leading to severe current leakage and a PCE of less than 1%. To address these challenges, we designed and synthesized the ATz‐benzodithiophene (BDT) copolymer **PBA‐T2** (Figure 1E).^[^ ^17^ ^]^ By incorporating a bulky alkylthienyl side chain into the BDT core, we improved solubility and miscibility, reduced aggregation, and facilitated the formation of a well‐separated and suitably blended morphology.^[^ ^17^ ^]^ This modification also suppressed lamellar formation,^[^ ^18^, ^19^, ^20^, ^21^ ^]^ and promoted a face‐on molecular orientation, which is favorable for efficient charge transport. However, the complex synthetic route of **PBA‐T2** results in high production costs, and the chemical instability of the BDT moiety at ambient temperature and under light irradiation poses significant limitations for its practical application in OPVs.^[^ ^22^ ^]^

In this study, we designed two copolymers, **PATz3T‐o6BO** and **PATz3T‐o6HD**, by incorporating the ATz unit and different alkyl groups into a terthiophene (three thiophene rings) structure (Figure 1E). Compared to **PATz4T‐o6OD**, which uses quaterthiophene (four thiophene rings) as donor units, these polymers are expected to exhibit a wavy structure throughout the polymer backbone due to the more stable conformation between adjacent thiophene rings. This backbone structure is anticipated to reduce aggregation by minimizing the overlap of π‐orbitals between polymer chains and promoting a favorable face‐on orientation for OPVs, without introducing the complex conformational BDT moieties.^[^ ^23^, ^24^, ^25^ ^]^ Indeed, the results of density functional theory (DFT) calculations of model dimers for **PATz3T‐o6BO** and **PATz3T‐o6HD** (Figure S5) indicate that these polymers have a wavy, twisted backbone with a 43.2° dihedral angle between the alkyl thiophene and the adjacent thiophene. This geometry is believed to suppress the effective overlap of π‐orbitals between the polymer chains, further reducing aggregation.

## 2 Results and Discussion

**Scheme 1:**
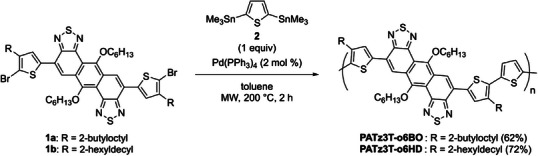
Synthesis of **PATz3T‐o6BO** and **PATz3T‐o6HD**.

First, monomers **1a** and **1b** were synthesized according to our previously reported procedure (see Supporting Information, Schemes S1,S2, and Figures S1,S2).^[^ ^15^, ^17^ ^]^ Subsequently, the target polymers, **PATz3T‐o6BO** and **PATz3T‐o6HD,** were successfully synthesized via Migita–Kosugi‐Stille polymerization yielding in 62% and 72%, respectively, as illustrated in Scheme 1 (Schemes S3,S4, and Figures S3,S4). Their number‐average molecular weights (*M*_n_) and polydispersity indices (*Đ*) were 45.8 and 32.5 kDa, 2.53 and 1.82, respectively (Figure S6). These polymers exhibited high solubility in chloroform and toluene even at room temperature.

**Figure 2:**
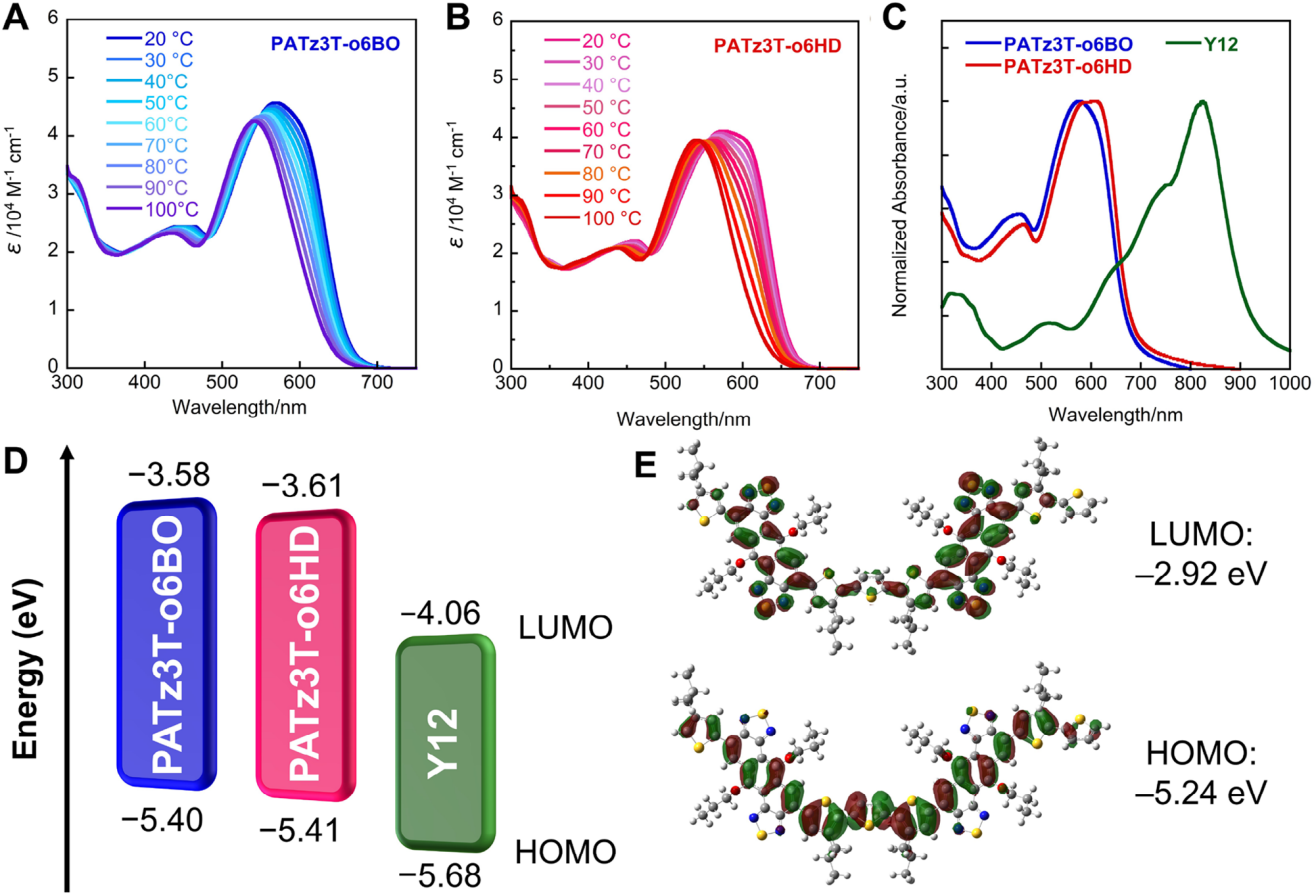
Temperature‐dependent UV–vis absorption spectra of (A) **PATz3T‐o6BO** and (B) **PATz3T‐o6HD** in chlorobenzene. (C) UV–vis absorption spectra of **PATz3T‐o6BO**, **PATz3T‐o6HD**, and **Y12** in thin film. (D) Energy diagram of **PATz3T‐o6BO**, **PATz3T‐o6HD**, and **Y12**. (E) HOMO/LUMO energy levels and geometries of the model compound of **PATz3T**, calculated using DFT at B3LYP/6‐311G(d).

Figure 2A–C show the UV–vis absorption spectra of **PATz3T‐o6BO** and **PATz3T‐o6HD**, with the corresponding data summarized in Table 1. Figure 2A,B display the temperature‐dependent absorption spectra of **PATz3T‐o6BO** and **PATz3T‐o6HD** in chlorobenzene, measured in 10 °C increments from 20 to 100 °C. At room temperature, **PATz3T‐o6BO** exhibited a maximum absorption (*λ*_max_) at 569 nm, while **PATz3T‐o6HD** showed a slightly red‐shifted absorption peak at 575 nm and a distinct vibronic shoulder at 606 nm. Upon heating the solution to 100 °C, both polymers exhibited a blue shift in their absorption spectra, indicating complete disaggregation in solution. These results suggest that both polymers exhibit strong aggregation tendencies while retaining good solubility. More specifically, the intermolecular interactions in **PATz3T‐o6HD** are stronger than those in **PATz3T‐o6BO**. Furthermore, in the thin‐film state, **PATz3T‐o6BO** exhibited a slightly red‐shifted absorption with a *λ*_max_ of 576 nm, while **PATz3T‐o6HD** exhibited a distinct absorption maximum that was more bathochromically shifted, at 607 nm. This indicates that the polymer backbone is well‐ordered in the solid state (Figure 2C). The calculated optical energy gaps (*E*_g_) for **PATz3T‐o6BO** and **PATz3T‐o6HD** were 1.82 and 1.80 eV, respectively, which are comparable to that of **PATz4T‐o6OD**.^[^ ^15^, ^16^, ^17^ ^]^

**Table 1:** Physicochemical properties of **PATz3T‐o6BO** and **PATz3T‐o6HD**.

Compound	*λ*_max, sol_ (nm)^a)^	*λ*_max, film_ (nm)^b)^	*E*_g_^opt^ (eV)^c)^	*E*_HOMO_ (eV)^d)^, ^e)^	*E*_LUMO_ (eV)^f)^
**PATz3T‐o6BO**	569	576	1.82	─5.40	─3.58
**PATz3T‐o6HD**	575	607	1.80	─5.41	─3.61

^a)^ Absorption maxima in chlorobenzene solution at room temperature.

^b)^ Absorption maxima in thin films.

^c)^ Optical energy gaps estimated from the absorption edge (*λ*_edge_).

^d)^ All potentials were calibrated with the standard ferrocene/ferrocenium redox couple (Fc/Fc^+^: *E* ^1/2^ = +0.08 V measured under identical conditions).

^e)^ Estimated by oxidation onset vs. Ag/Ag^+^; *E*_HOMO_ = −4.72−*E* ^ox^_onset_.

^f)^ Estimated by following equation: *E*_LUMO_ = *E*_HOMO_ + *E*_g_^opt^.

Cyclic voltammetry (CV) measurements were performed on the polymer thin films (Figure S7), and the results are also summarized in Table 1. The estimated HOMO/LUMO energy levels of **PATz3T‐o6BO** and **PATz3T‐o6HD** are −5.40/−3.58 eV and −5.41/−3.61 eV, respectively, compared to −5.39/−3.63 eV for **PATz4T‐o6OD** (Figure 2D).^[^ ^15^, ^17^ ^]^ This trend is consistent with the DFT calculations (Figure 2E); however, replacing thiophene with bithiophene has little impact on the energy levels. According to the DFT results, the LUMO coefficients of both **PATz3T‐o6R** and **PATz4T‐o6OD** are localized within the ATz core. Therefore, the substitution of thiophene with bithiophene is expected to have only a minor effect on the LUMO energy levels. In contrast, the electron‐donating ability of bithiophene is greater than that of thiophene, which is reflected in the HOMO levels (**PATz3T‐o6R**: HOMO = −5.24 eV; **PATz4T‐o6OD**: HOMO = −5.09 eV), as the HOMO is delocalized along the entire polymer backbone, as shown in the DFT calculations. We thus conclude that the slight change in the HOMO energy levels can plausibly be attributed to a reduced effective π‐conjugation length caused by an increased dihedral angle between thiophene units.^[^ ^26^, ^27^, ^28^ ^]^

**Figure 3:**
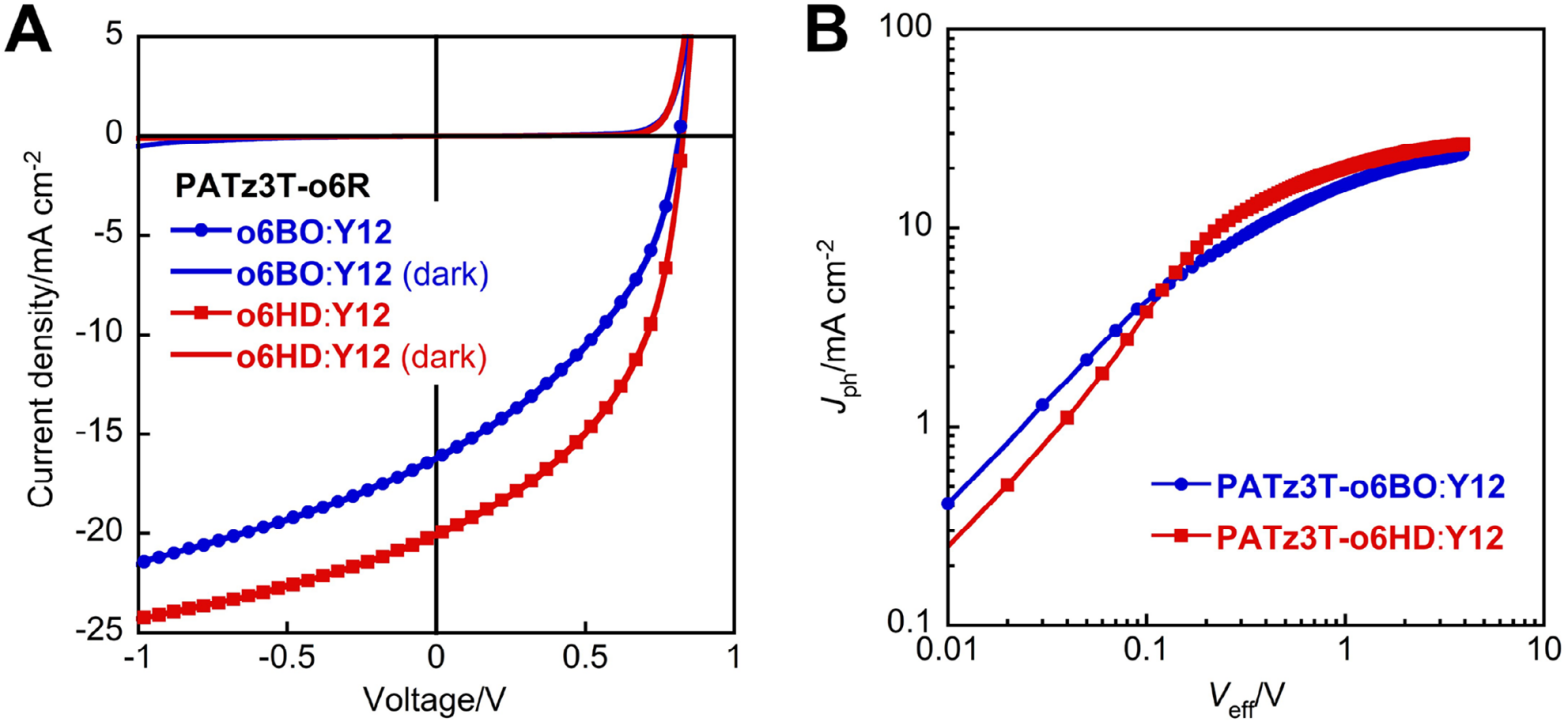
(A) Current density (*J*)–voltage (*V*) curves and (B) the *J*_ph_ versus *V*_eff_ curves of **PATz3T‐o6BO**:**Y12** and **PATz3T‐o6HD**:**Y12**‐based solar cells.

Next, to evaluate the photovoltaic properties of the synthesized polymers, conventional OPVs (Figure S8) with an ITO/(PEDOT:PSS)/**PATz3T‐o6R**:**Y12**/**PDINO**/Ag device configuration were fabricated and characterized (Figure 3A, and Tables 2, S1, and S2). Both **PATz3T‐o6BO** and **PATz3T‐o6HD** exhibited improved solubility, leading to the formation of uniform blended films with **Y12**. The **PATz3T‐o6BO**:**Y12**‐based cell exhibited a PCE of 5.34% with a *J*_sc_ of 16.21 mA cm^−2^, a *V*_oc_ of 0.82 V, and an FF of 0.40. In contrast, the **PATz3T‐o6HD**:**Y12**‐based cell achieved a PCE of 7.94% with a *J*_sc_ of 19.51 mA cm^−2^, a *V*_oc_ of 0.83 V, and an FF of 0.49, which is comparable to the previously reported polymer **PBA‐T2**.^[^ ^17^ ^]^ Under dark conditions, the current densities of both solar cells in the reverse bias region were significantly small. In contrast, under 1 sun illumination, steep slopes (i.e., low shunt resistance) were observed in the reverse bias region of the *J*‐*V* curves. These results suggest that poor interfacial morphology may lead to current leakage and surface recombination, resulting in a low FF.^[^ ^29^ ^]^ To investigate the effect of side chains (the alkyl groups) on the *J*_sc_ and FF, the overall efficiency of the exciton dissociation and charge collection (*P*_diss_) was examined by plotting photocurrent (*J*_ph_) against effective applied voltage (*V*_eff_) (Figure 3B).^[^ ^30^ ^]^ The **PATz3T‐o6HD**:**Y12**‐based cell exhibited a *P*_diss_ of 74.5%, which is higher than the **PATz3T‐o6BO**:**Y12**‐based cell (*P*_diss_ = 65.9%), indicating better exciton dissociation and charge collection in the **PATz3T‐o6HD**:**Y12**‐based device. We also investigated the extent of trap‐assisted and bimolecular recombination, as shown in Figure S9.^[^ ^31^, ^32^ ^]^ Trap‐assisted recombination can be expressed by the equation *V*_oc_ ∝ *nkT/q*ln(*P*_light_), which illustrates the relationship between *V*_oc_ and *P*_light_. On the other hand, bimolecular recombination is expressed by the equation *J*_sc_ ∝ (*P*_light_)*^α^*, which depicts the relationship between *J*_sc_ and *P*_light_. The smaller *n* value (1.16 *kT*/*q*) and higher *α* value (0.934) of the **PATz3T‐o6HD**:**Y12**‐based device, compared to the **PATz3T‐o6BO**:**Y12**‐based cell (*n* = 1.21 *kT*/*q* and *α* = 0.921), suggest that both trap‐assisted and bimolecular recombination were effectively suppressed in the **PATz3T‐o6HD**:**Y12** system. This suppression contributed to the relatively higher *J*_sc_ and FF observed. However, both the **PATz3T‐o6BO** and **PATz3T‐o6HD**‐based devices showed low *α* values (below 0.94), indicating low charge collection efficiency, which ultimately hindered improvements in *J*_sc_ and FF than anticipated. As shown in Figure S10, the hole and electron mobility of the **PATz3T‐o6BO** and **PATz3T‐o6HD**‐based devices, estimated from the space‐charge limited current (SCLC) method, were 1.79/0.79 × 10^−4^ and 1.94/0.95 × 10^−4^ cm^2^ V^−1^ s^−1^, respectively. These results further support the limited *J*_sc_ and FF, as the unbalanced mobility may lead to significant bimolecular recombination.

**Table 2:** The best solar cell performances of **PATz3T‐o6BO** and **PATz3T‐o6HD**‐based devices.^a)^

Active Layer	*J*_sc_ (mA cm^−2^)	*V*_oc_ (V)	FF	PCE (%)
**PATz3T‐o6BO:Y12**	16.21 (15.83 ± 0.26)	0.82 (0.81 ± 0.00)	0.40 (0.41 ± 0.01)	5.34 (5.29 ± 0.04)
**PATz3T‐o6HD:Y12**	19.51 (18.36 ± 0.79)	0.83 (0.82 ± 0.01)	0.49 (0.50 ± 0.02)	7.94 (7.51 ± 0.25)

^a)^ Average values and standard deviations are shown in parentheses.

**Figure 4:**
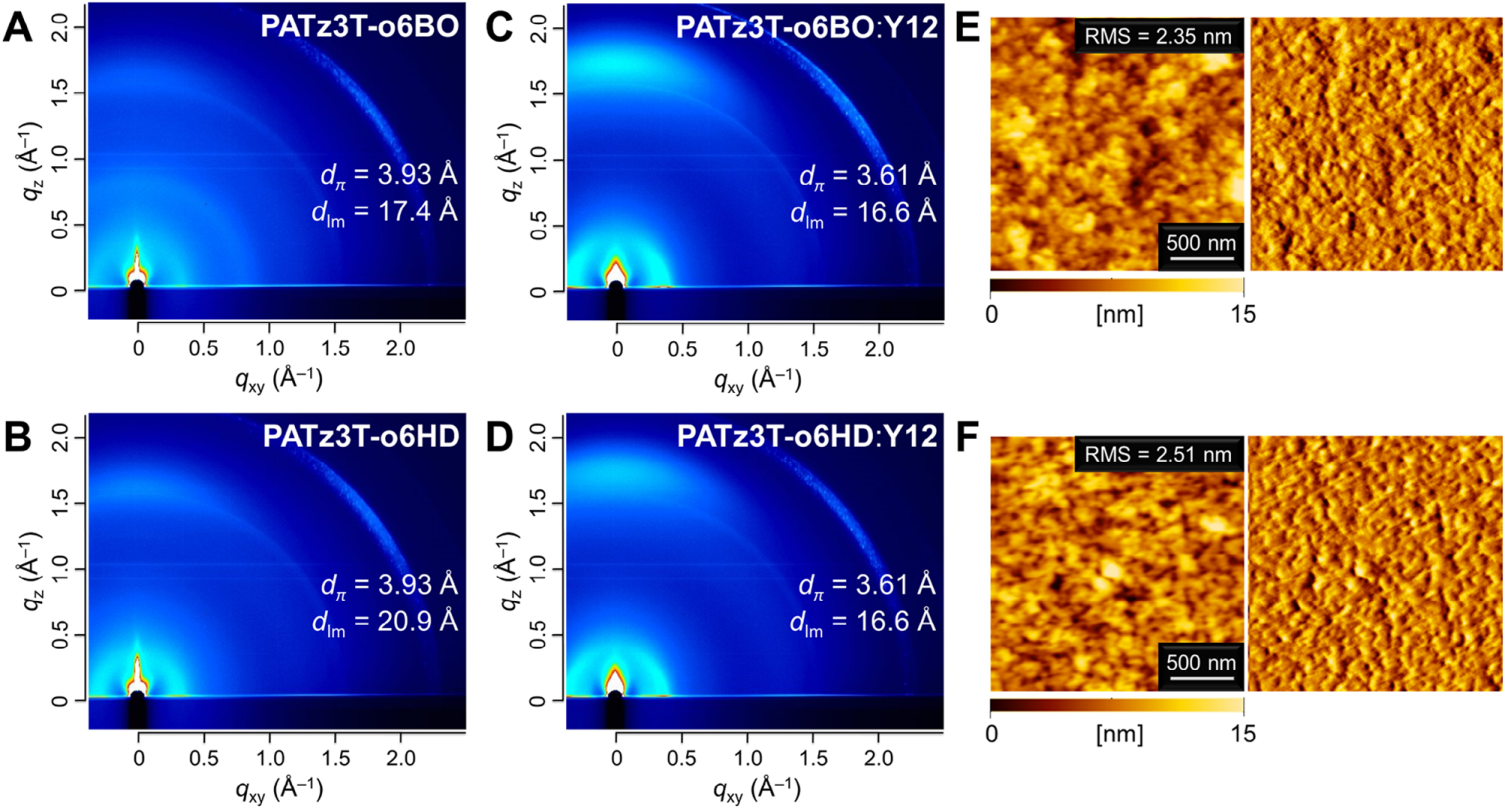
GIWAXS images of (A) neat **PATz3T‐o6BO** and (B) neat **PATz3T‐o6HD** films; (C) **PATz3T‐o6BO**:**Y12** and (D) **PATz3T‐o6HD**:**Y12** blended films. AFM images of (E) **PATz3T‐o6BO** and (F) **PATz3T‐o6HD** blended films with **Y12**: (left) topographic and (right) error‐signal images.

Additionally, grazing incidence wide‐angle X‐ray scattering (GIWAXS) measurements were conducted to investigate the correlation between thin‐film structure and photovoltaic performance (Figure 4A–D). Both **PATz3T‐o6BO** and **PATz3T‐o6HD** exhibited distinct π‐stacking diffraction along the *q*_z_ axis and lamellar diffraction along the *q*_xy_ axis, indicating the formation of a face‐on orientation, which is favorable for high‐performance OPVs. The π‐stacking distance of **PATz3T‐o6BO** (*d*_π_ = 3.93 Å) is identical to that of **PATz3T‐o6HD** (*d*_π_ = 3.93 Å). However, the lamellar distance (*d*_lm_) of **PATz3T‐o6HD** (20.9 Å) is larger than that of **PATz3T‐o6BO** (17.4 Å), which can be attributed to the difference in their alkyl chain lengths. In the blended films with **Y12**, both **PATz3T‐o6BO** and **PATz3T‐o6HD** exhibited identical lamellar and π‐stacking diffraction peaks, with *d*_lm_ of 16.6 Å and *d*_π_ of 3.61 Å, respectively, both adopting a face‐on orientation. Figure 4E,F display atomic force microscopy (AFM) images of films of **PATz3T‐o6BO** and **PATz3T‐o6HD** blended with **Y12**. These films exhibited similar surface morphologies, with root‐mean‐square (RMS) roughness values of 2.35 and 2.51 nm, respectively. Therefore, we speculated that the higher photovoltaic performance of **PATz3T‐o6HD**:**Y12**‐based OPV may be due to their higher domain purity.^[^ ^33^ ^]^ However, relatively large phase separation, with RMS greater than 2 nm, was observed in both **PATz3T‐o6BO**:**Y12** and **PATz3T‐o6HD**:**Y12** blended films. In general, the optimal p/n ratio for typical wide‐bandgap polymer donors and low‐bandgap SMAs, such as **Y12**, is around 1:1.2 to achieve favorable blend morphology.^[^ ^7^ ^]^ However, in our polymer systems, the optimal p/n ratio is 1:2, indicating a significantly higher SMA content compared to conventional polymer donor–SMA systems. This suggests that an appropriate blend morphology cannot be achieved without the addition of excess SMAs. Indeed, **PATz3T**:**Y12**‐based devices with p/n ratios of 1:1 or 1:1.2 exhibited lower *J*_sc_ and FF (Tables S1 and S2). We therefore conclude that the pronounced phase separation observed in these systems arises from the intrinsically strong aggregation tendency of **PATz3T‐o6BO** and **PATz3T‐o6HD**, which hinders the formation of an ideal blend morphology with **Y12**. As a result, efficient charge separation and transport are suppressed in the **PATz3T‐o6BO**:**Y12** and **PATz3T‐o6HD**:**Y12**‐based devices, leading to limited *J*_sc_ and FF.^[^ ^34^, ^35^ ^]^

## 3 Conclusion

In summary, we synthesized the ATz‐terthiophene copolymers **PATz3T‐o6BO** and **PATz3T‐o6HD** as high‐performance polymer donors for application in OPVs. By using terthiophene as a less symmetric donor unit, the resulting polymers exhibited a wavy backbone structure, which reduced aggregation, enhanced solubility, and improved molecular ordering. Consequently, these polymers produced homogeneous blended films with **Y12**, and the solar cells based on **PATz3T‐o6HD** achieved a maximum PCE of 7.94%. GIWAXS measurements revealed that both polymers formed a favorable face‐on orientation; however, they still exhibited relatively large phase separation due to strong aggregation, which may have limited their photovoltaic performance. However, the aggregation strength of these polymers can likely be further controlled through additional side‐chain engineering in the future. In addition, our ATz‐based polymers exhibit strong aggregation and promote an ordered molecular structure due to the presence of the rigid and π‐extended ATz framework, even though the polymers lack a highly planar backbone conformation. This highlights a key advantage of incorporating the ATz unit into the polymer backbone. Overall, the terthiophene‐based strategy provides an effective approach to enhancing the solubility of π‐extended polymers and offers valuable insights into the molecular design of high‐performance polymer donors for OPV applications.

## Supporting Information

Additional references are cited in the Supporting Information. Full characterization data, including ^1^H and ^13^C{^1^H} NMR spectra of all new compounds and polymers, details of theoretical calculations, physicochemical properties, device characteristics, light‐intensity dependence of the solar cells, and SCLC‐derived hole and electron mobilities, are provided in the Supporting Information.

## Conflict of Interests

The authors declare no conflicts of interest.

## Supporting information



Supporting Information

## Data Availability

The data that support the findings of this study are available in the Supporting Information of this article.
