# Supplementary material for: Alkoxy‐Substituted Anthrabis(Thiadiazole)‐Terthiophene Copolymers for Organic Photovoltaics: A Unique Wavy Backbone Enhances Aggregation, Molecular Order, and Device Efficiency
Source: Chem Asian J. 2025 Jun 23;20(18):e00678. doi: 10.1002/asia.202500678 (PMC12450037; doi:10.1002/asia.202500678)
Supplement: Supplementary file 1 — Supporting Information [file ASIA-20-e00678-s001.pdf]

Electronic Supporting Information for:

Alkoxy-Substituted Anthrabis(Thiadiazole)-Terthiophene Copolymers for Organic Photovoltaics: A Unique Wavy Backbone Enhances Aggregation, Molecular Order, and Device Efficiency

Yi Yan,[a] Hiroki Mori,*[b] Tomoki Yoshino,[a] Ryuki Inami,[a]

Jiaxin Chang,[a] Junqing Gao,[a] and Yasushi Nishihara*[b]

[a]*Graduate School of Environmental, Life, Natural Science and Technology, Okayama University, 3-1-1 Tsushimanaka, Kita-ku, Okayama 700-8530, Japan.*

[b]*Research Institute for Interdisciplinary Science, Okayama University, 3-1-1 Tsushimanaka, Kita-ku, Okayama 700-8530, Japan.*

Phone: +81-86-251-7855

Fax: +81-86-251-7855

E-mail: h-mor@okayama-u.ac.jp

E-mail: ynishiha@okayama-u.ac.jp

General

All reactions were carried out under an Ar atmosphere using standard Schlenk techniques. Glassware was dried in an oven at 130 ºC and then heated under reduced pressure prior to use. Dehydrated tetrahydrofuran (THF), *N*,*N*-dimethylformamide (DMF), and toluene were purchased from Kanto Chemicals Co., Inc. Thin-layer chromatography (TLC) analyses were conducted using Merck precoated TLC plates (silica gel 60 GF_{254}, 0.25 mm). Silica gel column chromatography was carried out using Silica gel 60 N (spherical, neutral, 40-100 μm) from Kanto Chemicals Co., Inc. The ^{1}H, $^{13}C\{^{1}H\}$ NMR spectra were recorded on Varian 400-MR (400 MHz), Varian NMR System PS600, and JEOL JNMECZ600R (600 MHz) spectrometers. Chemical shifts (δ) are reported in parts per million relative to $CDCl_3$ at 7.26 ppm for ^{1}H and at 77.16 ppm for $^{13}C\{^{1}H\}$ NMR measurements, respectively. Infrared spectra were recorded using a Shimadzu IRPrestige-21 spectrophotometer. Elemental analyses were carried out with a Perkin-Elmer 2400 CHN elemental analyzer at Okayama University. Polymerizations were performed using a Biotage initiator^{+} microwave reactor. Molecular weights of the polymers were determined by gel-permeation chromatography (GPC) using a TOSOH HLC-8321GPC/HT and TSKgel GMH_{HR}-H HT column, with polystyrene standards and *o*-dichlorobenzene (*o*-DCB) as the eluent at 140 °C.

4,10-Bis(5-bromo-4-(2-butyloctyl)thiophene-2-yl)-6,12-bis(hexyloxy)anthra[1,2-*c*:5,6-*c*']bis([1,2,5]thiadiazole) (**1a**)[1] and 2,5-bis(trimethylstannyl)thiophene (**2**)[2] were synthesized according to reported procedures. Other chemicals were used without further purification unless otherwise indicated.

Experimental Procedures and Copies of ^{1}H, $^{13}C\{^{1}H\}$ NMR Charts for the New Compounds

Scheme S1. Synthesis of 4,10-bis(4-(2-hexyldecyl)thiophene-2-yl)-6,12-bis(hexyloxy)anthra[1,2-*c*:5,6-*c'*]bis([1,2,5]thiadiazole) (**5**)

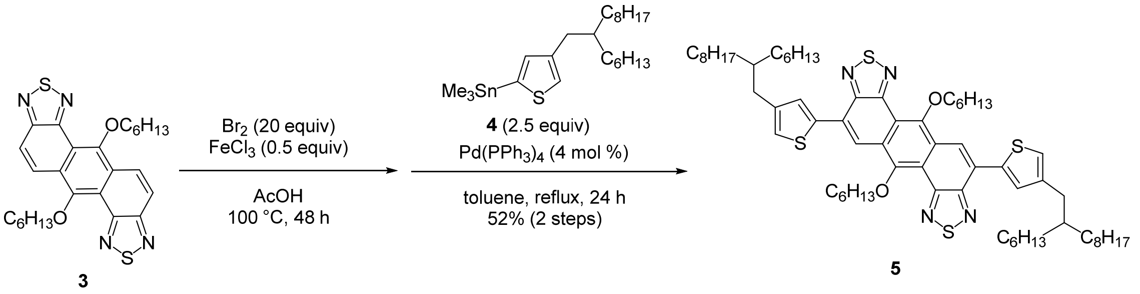

Under ambient atmosphere, a 50 mL Schlenk tube was charged with **3** (252 mg, 0.51 mmol), acetic acid (40 mL), iron(III) chloride (41.4 mg, 0.26 mmol, 0.5 equiv), and bromine (526 μL, 10.2 mmol, 2 equiv). The mixture was heated and stirred at 100 °C for 48 hours. After cooling to room temperature, the reaction was quenched by adding water, and the precipitate was collected by filtration. The resulting brown solid was washed with methanol and used in the subsequent reaction.

Under an argon atmosphere, the previously synthesized brominated compound, 4-(2-hexyldecyl)-2-trimethylstannylthiophene **4** (601 mg, 1.3 mmol, 2.5 equiv), anhydrous toluene (20 mL), and tetrakis(triphenylphosphine)palladium(0) (23.6 mg, 4 mol%) were added to a 50 mL Schlenk flask and heated to reflux for 24 h. After cooling the reaction mixture to room temperature, water was added to quench the reaction, and the mixture was extracted with chloroform. The organic layer was washed with 1 M potassium fluoride aqueous solution and brine, then dried over anhydrous magnesium sulfate. After filtration, the solvent was removed using a rotary evaporator. The crude product was purified by silica gel column chromatography (hexane/CH_2Cl_2 = 5:1), yielding the target compound **5** as an orange solid with an overall yield of 52% (291 mg, 0.26 mmol) over two steps. Mp 99–102°C. FT-IR (KBr, cm^{-1}): 2924 (s), 2851 (s), 1587 (s), 1466 (s), 1378 (s), 1312 (s), 1263 (s), 1060 (m), 866 (m), 723 (m). ^{1}H NMR (600 MHz, $CDCl_3$, rt): δ 8.79 (s, 2H), 8.14 (s, 2H), 7.06 (s, 2H), 4.28 (t, J = 6.6 Hz, 4H), 2.68 (d, J = 6.0 Hz, 4H), 2.25–2.21 (m, 4H), 1.84–1.73 (m, 6H), 1.53–1.25 (m, 56H), 1.00–0.97 (m, 6H), 0.89–0.85 (m, 12H). $^{13}C\{^{1}H\}$ NMR (151 MHz, $CDCl_3$, rt): δ 153.54, 152.06, 150.01, 143.13, 138.95, 130.27, 128.10, 126.29, 122.57, 122.42, 119.66, 75.61, 38.91, 35.14, 33.36, 33.34, 32.01, 31.94, 31.92, 30.38, 30.08, 29.75, 29.67, 29.37, 26.64, 26.61, 26.24, 22.81, 22.71, 22.68, 14.20, 14.14, 14.12. Anal. Calcd for $C_{66}H_{98}N_4O_2S_4$: C, 71.56; H, 8.92; N, 5.06%. Found: C, 71.49; H, 8.73; N, 5.10%.

Scheme S2. Synthesis of 4,10-bis(5-bromo-4-(2-hexyldecyl)thiophene-2-yl)-6,12-bis(hexyloxy)anthra[1,2-*c*:5,6-*c*']bis([1,2,5]thiadiazole) (**1b**)

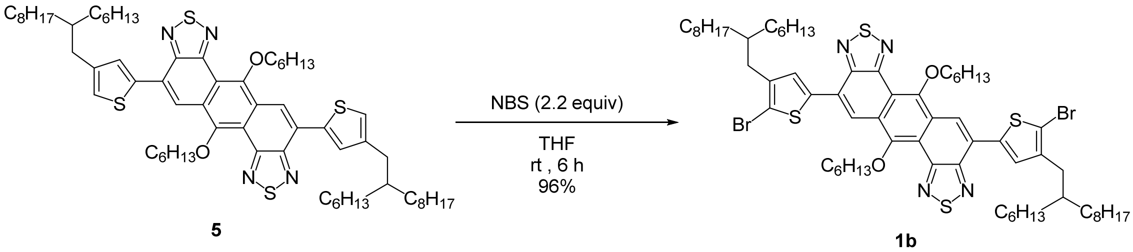

Under an argon atmosphere, **5** (108 mg, 0.097 mmol), anhydrous THF (25 mL), and *N*-bromosuccinimide (40.8 mg, 0.21 mmol, 2.2 equiv) were added to a 50 mL Schlenk flask and stirred for 6 h. The reaction was then quenched by adding water and extracted using chloroform. The organic layer was washed with brine and dried over anhydrous magnesium sulfate. After filtration, the solvent was removed using a rotary evaporator. Further recrystallization using ethyl acetate afforded the target compound **1b** as an orange solid in 96% yield (119 mg, 0.093 mmol). Mp 105–107°C. FT-IR (KBr, cm^{-1}): 2955 (s), 2924 (s), 2854 (s), 2359 (m), 1465 (m), 1431 (m), 1379 (m), 1318 (m), 1264 (m), 1040 (m), 844 (m), 813 (m), 799 (m), 724 (s). ^{1}H NMR (600 MHz, $CDCl_3$, rt): δ 8.70 (s, 2H), 7.97 (s, 2H), 4.25 (t, J = 6.0 Hz, 4H), 2.62 (d, J = 7.2 Hz, 4H), 2.22–2.20 (m, 4H), 1.84–1.80 (m, 6H), 1.57–1.24 (m, 56H), 1.01–0.99 (m, 6H), 0.88–0.85 (m, 12H). $^{13}C\{^1H\}$ NMR (151 MHz, $CDCl_3$, rt): δ 152.94, 151.75, 149.82, 142.39, 129.59, 127.90, 125.07, 121.90, 119.44, 111.91, 75.39, 38.76, 34.50, 33.57, 33.54, 32.30, 32.14, 32.11, 30.57, 30.30, 29.97, 29.87, 29.58, 26.74, 26.71, 26.59, 23.10, 22.91, 22.87, 14.48, 14.33, 14.29. Anal. Calcd for $C_{66}H_{96}Br_2N_4O_2S_4$: C, 62.64; H, 7.65; N, 4.43%. Found: C, 62.49; H, 7.37; N, 4.32%.

Scheme S3. Synthesis of **PATz3T-o6BO**

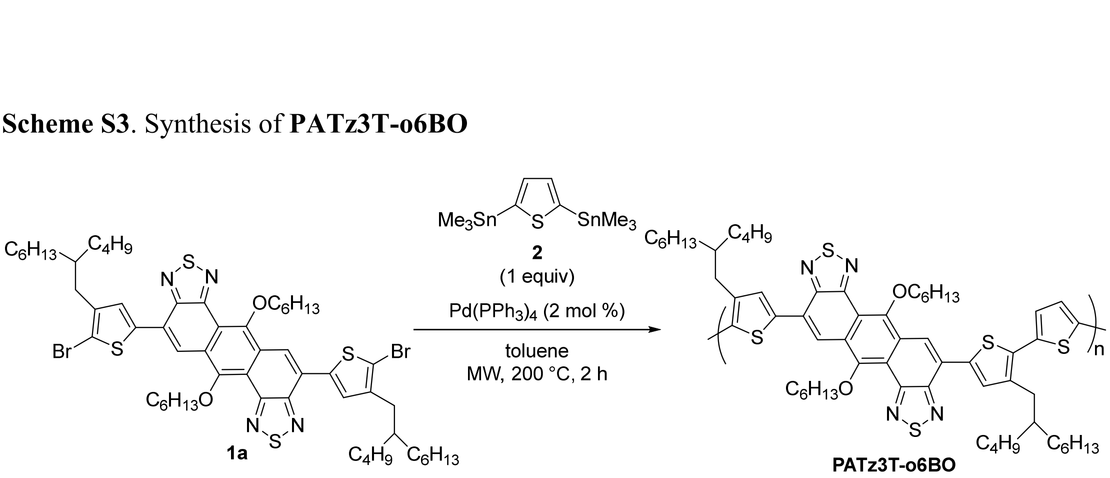

In a 5 mL microwave reactor vessel, **1a** (57.7 mg, 0.05 mmol), **2** (20.5 mg, 0.05 mmol, 1 equiv),

and $Pd(PPh_3)_4$ (1.2 mg, 1.0 μmol, 2 mol %) were added, and the vessel was sealed under argon gas. Then, anhydrous toluene (2 mL) was added, and the mixture was heated at 200 °C for 2 h using a microwave reactor. After the reaction, the mixture was added to 100 mL of a 5% hydrochloric acid/methanol solution and stirred at room temperature for 3 hours. The resulting precipitate was collected by filtration and then purified by Soxhlet extraction with methanol, hexane, and chloroform solvents in sequence. The chloroform-soluble components were concentrated, and reprecipitation with methanol resulted in the desired compound **PATz3T-o6BO**, obtained as a uniform purple film with metallic luster in 62% yield (33.4 mg). ^1H NMR (600 MHz, *o*-dichlorobenzene-d_4, 80 °C): δ 8.81 (brs), 8.31 (brs), 4.29 (brs), 2.92 (brs), 2.23 (brs), 1.97 (brs), 1.81 (brs), 0.76-1.44 (brs). GPC (*o*-DCB, 140 ºC): M_n = 45.8 kDa, M_w = 114.6 kDa, PDI = 2.53, DP_n = 43. Anal. Calcd for $C_{62}H_{84}N_4O_2S_5$: C, 69.10; H, 7.86; N, 5.20%. Found: C, 69.11; H, 7.55; N, 5.03%.

Scheme S4. Synthesis of **PATz3T-o6HD**

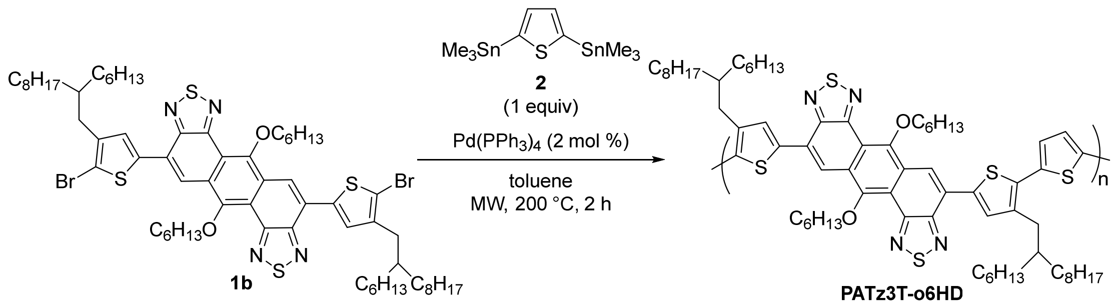

Monomers **1b** (63.3 mg, 0.05 mmol) and **2** (20.5 mg, 0.05 mmol, 1 equiv), tetrakis(triphenylphosphine)palladium(0) ($Pd(PPh_3)_4$, 1.2 mg, 2 μmol), and toluene (2 mL) were subjected to the polymerization procedure, and sequential Soxhlet extraction with the same solvents used for **PATz3T-o6BO** was employed to obtain **PATz3T-o6HD** (42.8 mg, 72%) as a metallic purple solid. ^1H NMR (600 MHz, *o*-dichlorobenzene-d_4, 80 °C): δ 8.74 (brs), 8.24 (brs), 4.22 (brs), 2.86 (brs), 2.18 (brs), 1.90 (brs), 1.74 (brs), 0.62-1.38 (brs). GPC (*o*-DCB, 140 ºC): M_n = 32.5 kDa, M_w = 59.1 kDa, PDI = 1.82, DP_n = 27. Anal. Calcd for $C_{70}H_{100}N_4O_2S_5$: C, 70.66; H, 8.47; N, 4.71%. Found: C, 70.90; H, 8.31; N, 4.68%.

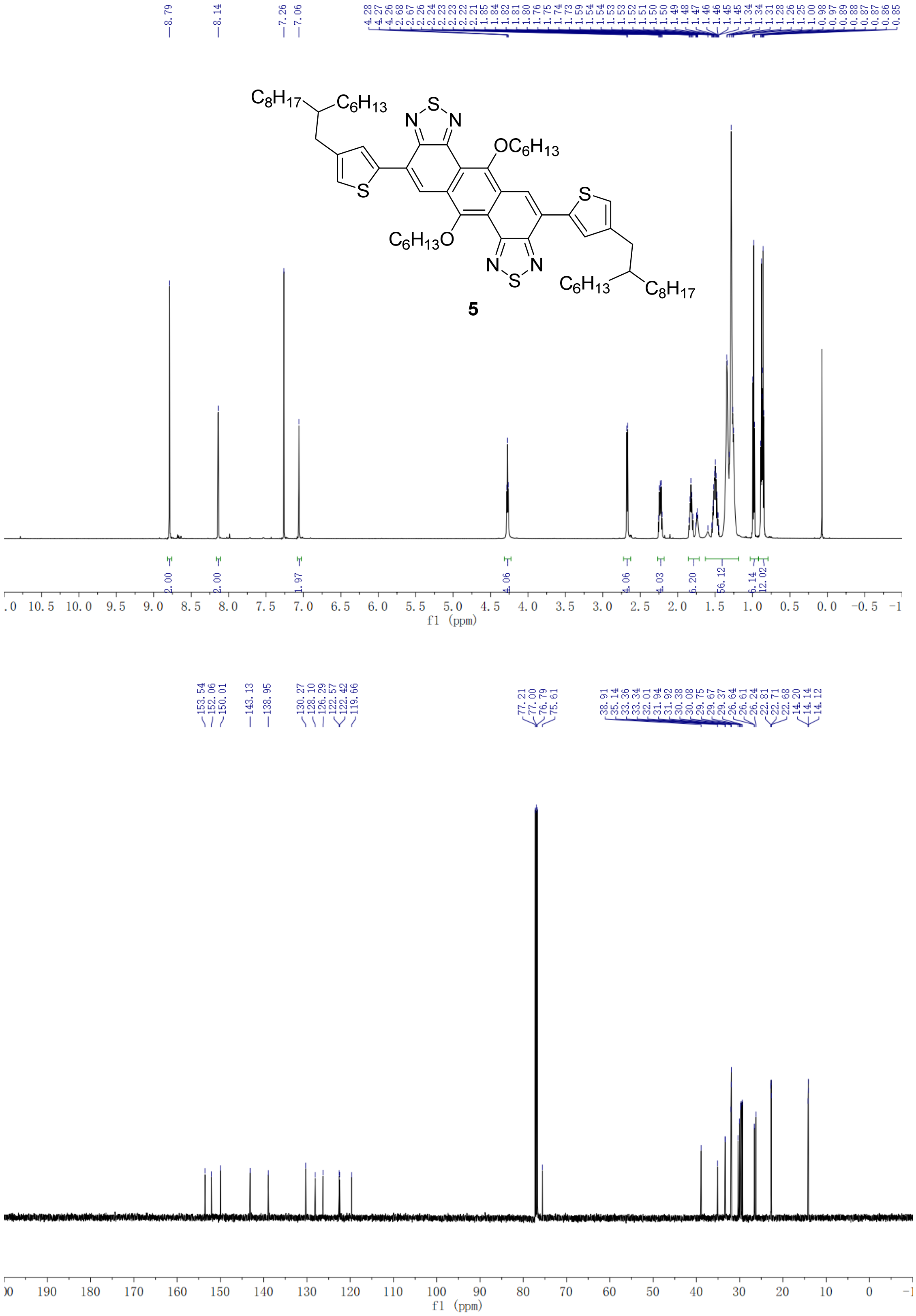

Figure S1. The ^{1}H (600 MHz) and $^{13}C\{^{1}H\}$ (151 MHz) NMR spectra of **5** ($CDCl_3$, rt).

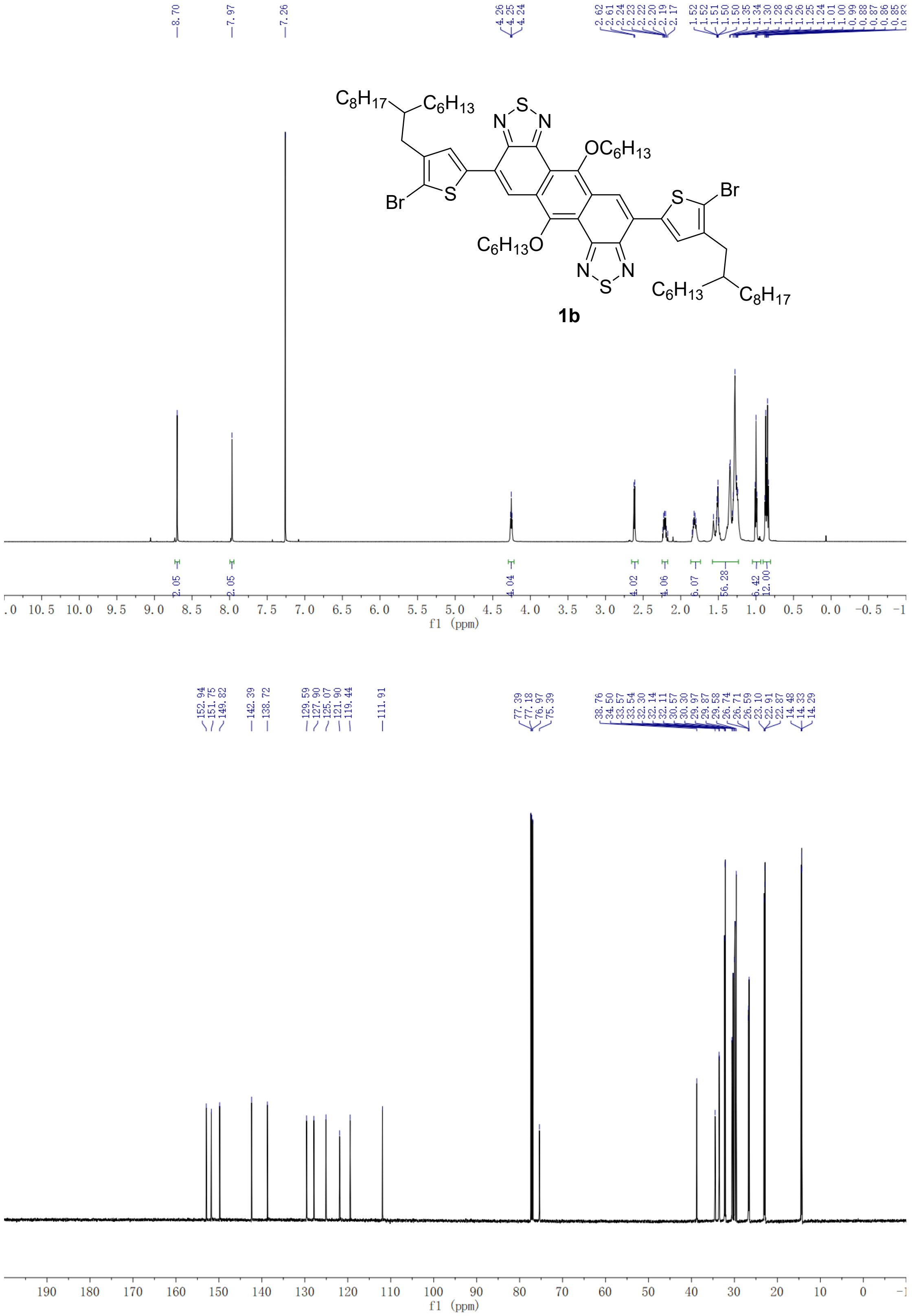

Figure S2. The ^{1}H (600 MHz) and $^{13}C\{^{1}H\}$ (151 MHz) NMR spectra of **1b** ($CDCl_3$, rt).

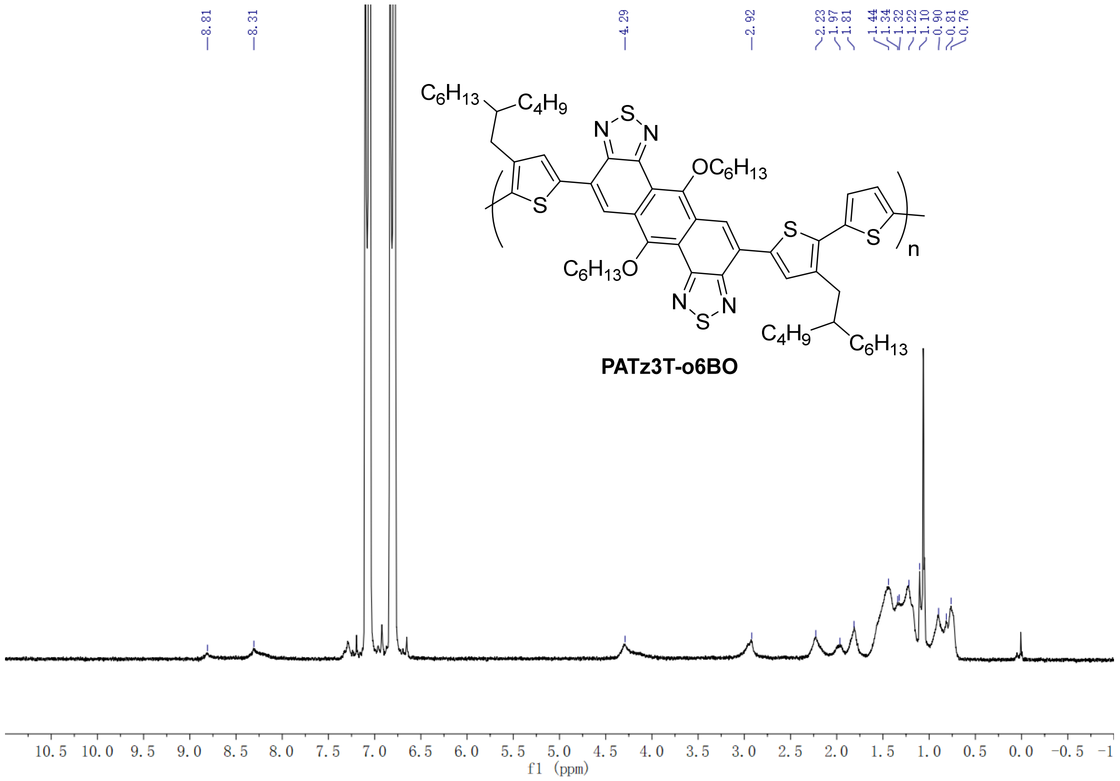

Figure S3. The ^{1}H NMR (600 MHz) spectrum of **PATz3T-o6BO** (*o*-dichlorobenzene-d_4, 80 °C).

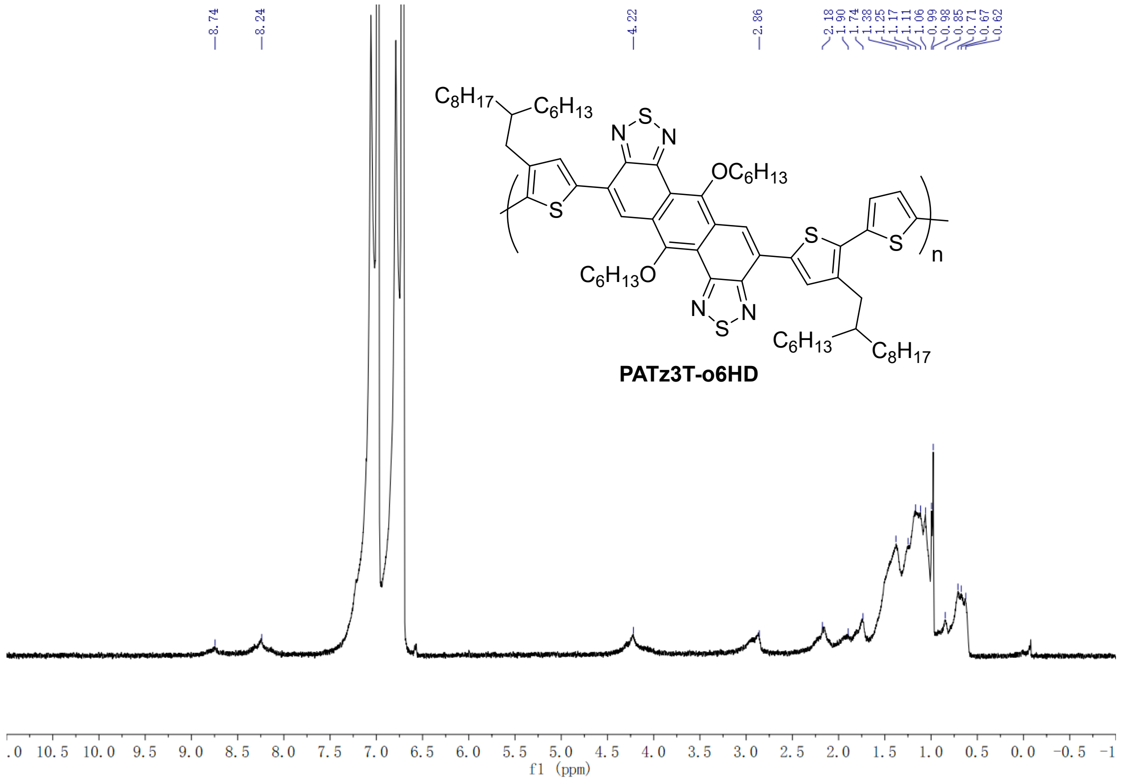

Figure S4. The ^{1}H NMR (600 MHz) spectrum of **PATz3T-o6HD** (*o*-dichlorobenzene-d_4, 80 °C).

Instrumentation and Theoretical Calculations

UV–vis absorption spectra were measured using a Shimadzu UV-2600i UV−vis spectrophotometer equipped with an ISR-2600Plus integrating sphere attachment. Cyclic voltammograms (CVs) were recorded in acetonitrile containing tetrabutylammonium hexafluorophosphate ($TBAPF_6$, 0.1 M) as the supporting electrolyte at a scan rate of 100 mV/s using a CHI-600B Electrochemical Analyzer. The working electrode was a Pt electrode (surface area: A = 0.071 cm^2, BAS), the reference electrode was an Ag/Ag^+ (Ag wire in 0.01 M $AgNO_3$/0.1 M $TBAPF_6/CH_3CN$), and the counter electrode was a Pt wire. Polymer films were prepared by drop casting from their chlorobenzene solutions onto the working electrode. All potentials were calibrated using a standard ferrocene/ferrocenium redox couple (Fc/Fc^+: $E_{1/2}$ = +0.08 V measured under identical conditions). Dynamic force-mode atomic force microscopy (AFM) was carried out using a SPA 400-DFM (SII Nano Technologies). Grazing incidence wide-angle X-ray scattering (GIWAXS) analysis was carried out at SPring-8 on beamline BL13XU. GIWAXS patterns were recorded with a 2D image detector (Pilatus 300 K) following fixed-angle irradiation (0.12°) through a Huber diffractometer using X-ray energy of 12.39 keV (λ = 1 Å). Polymer films and blended films with **Y12** were fabricated by spin-coating onto (PEDOT:PSS)-treated ITO substrates.

Geometry optimization and normal-mode calculations were performed at the B3LYP/6-311G(d) level using the Gaussian 09 Revision D.01 program package.[3] Density functional theory (DFT) calculations were performed on its dimer structure. To simplify the DFT calculations, *n*-propyl and isobutyl groups were used in place of the alkyl chains.

Fabrication of Conventional OPVs, Hole-Only and Electron-Only Devices

Conventional bulk-heterojunction solar cells were fabricated as follows. *N*,*N*′-Bis(*N*,*N*-dimethylpropan-1-amine oxide)perylene-3,4,9,10-tetracarboxylic diimide (**PDINO**) and 2,2′-((2*Z*,2′*Z*)-((12,13-bis(2-butyloctyl)-3,9-diundecyl-12,13-dihydro-[1,2,5]thiadiazolo[3,4-*e*]thieno-[2″,3″:4′,5′]thieno[2′,3′:4,5]-pyrrolo[3,2-*g*]thieno[2′,3′:4,5]thieno[3,2-*b*]indole-2,10-diyl)bis-(methanylylidene))bis(5,6-difluoro-3-oxo-2,3-dihydro-1*H*-indene-2,1-diylidene))dimalononitrile (**Y12**) were purchased from Ossila Ltd., and used as received. ITO substrates (Geomatec Co. Ltd., thickness = 150 nm, sheet resistance < 12 Ω sq^{-1}, transmittance at λ = 550 nm ≥ 85%) were cleaned successively by ultrasonication in neutral detergent, deionized water, acetone, and 2-propanol at room temperature, followed by hot 2-propanol for 10 min. The cleaned ITO substrates were then treated with UV-ozone for 20 min. The precleaned ITO substrates were spin-coated with poly(3,4-ethylenedioxythiophene):polystyrene sulfonate (PEDOT:PSS) (Clevios P VP AI 4083, Heraeus) at 4000 rpm for 30 sec using a 0.45 µm PVDF syringe filter. After being baked at 150 °C for 15 min

in air, the substrates were immediately transferred to a nitrogen-filled glove box. The active layer containing **Y12** was deposited by spin-coating at 1000 rpm for 30 sec from a solution containing the polymer sample (4.17 mg/mL) and **Y12** (8.34 mg/mL) in chlorobenzene. The solution was heated at 60 °C for 1 h, then spin-coated onto the substrate at room temperature. The optimal p/n ratios for the **PATz3T-o6BO**:**Y12** and **PATz3T-o6HD**:**Y12** blends were 1:2 (w/w). 1,8-Diiodooctane (DIO, 0.5 vol%) was used as the optimal solvent additive. After being dried under reduced pressure, the **PDINO** (2 mg/mL in a 6:4 mixture of EtOH and 2,2,2-trifluoroethanol (TFE))[4] layer was spin-coated at 3000 rpm for 30 sec on top of the active layer. An Ag (100 nm) electrode was then deposited under high vacuum (~5×10^{-5} Pa) using a shadow mask. The active area of all devices was 0.16 cm^2.

The characteristics of the solar cell devices were measured through a 4 × 4 mm photomask using a Keithley 2401 semiconductor analyzer. These devices were illuminated with an Xe lamp (Bunkokeiki OTENTO-SAN III type G2) at room temperature, under a nitrogen atmosphere, and with AM 1.5 G simulated solar irradiation at 100 $mWcm^{-2}$. The light intensity was calibrated using a standard silicon solar cell (Bunkokeiki, BS-520BK).

Hole-only devices were fabricated using the following steps. ITO substrates were cleaned, and a (PEDOT:PSS) layer was deposited in the same manner as described above. The active layer, containing **Y12**, was deposited in the same manner as outlined earlier. The thickness of the active layer was approximately 100-110 nm for both **PATz3T-o6BO**:**Y12** and **PATz3T-o6HD**:**Y12**. After the active layer was dried under reduced pressure, MoO_3 (6 nm) was deposited as a cathode interlayer, followed by the deposition of Ag (100 nm) to form a 0.16 cm^2 area. Both layers were deposited under high vacuum (~5×10^{-5} Pa) through a shadow mask. For electron-only devices, a ZnO precursor solution was prepared via the hydrolysis of $Zn(OAc)_2 \cdot 2H_2O$.[5] ITO substrates were cleaned in the same manner for the hole-only devices. The precleaned ITO substrates were spin-coated with 0.4 M ZnO precursor solution at 4000 rpm for 30 sec, then immediately baked at 200 °C for 1 h in air. After gradually cooling to room temperature, the substrates were rinsed with acetone and 2-propanol at room temperature, followed by a 5 min wash in hot 2-propanol. The substrates were then dried and immediately transferred to a nitrogen-filled glove box. The active layer with **Y12** was deposited in the same manner as described earlier. The thickness of the active layer was approximately 100-110 nm for both **PATz3T-o6BO**:**Y12** and **PATz3T-o6HD**:**Y12**. A **PDINO** solution (2 mg/mL in a 6:4 mixture of EtOH and 2,2,2-trifluoroethanol (TFE)) was prepared and spin-coated at 3000 rpm for 30 sec on top of the active layer. After the active layer was dried under reduced pressure, an Ag (100 nm) electrode was deposited over the device area (0.16 cm^2)

through a shadow mask under high vacuum (~5 × 10^{-5} Pa). The thickness of the active layer was measured using an AlphaStep® IQ surface profiler (KLA Tencor).

The current density−voltage (*J–V*) characteristics were measured in the dark under a nitrogen atmosphere using a Keithley 2401 semiconductor analyzer. Voltage sweeps were performed in the range of 0–7 V. Hole and electron mobilities were estimated by fitting the *J–V* curve using the space charge limited current (SCLC) equation: $J = (9/8)\varepsilon_0\varepsilon_r\mu(V^2/L^3)$ where ε_0 is the permittivity of free space, ε_r is the dielectric constant of the polymer, μ is the hole or electron mobility, L is the thickness of the active layer, and V is the voltage drop across the device ($V = V_{appl} - V_{bi}$). Here, V_{appl} is the applied voltage, and V_{bi} is the offset voltage.

The Overall Efficiencies (P_{diss}) of the Exciton Dissociation and Charge Collection

The overall efficiencies (P_{diss}) of the exciton dissociation and charge collection were investigated by plotting the photocurrent (J_{ph}) against the effective applied voltage (V_{eff}).[6] The J_{ph} was obtained from the equation $J_{ph} = J_L - J_D$, where J_D and J_L stand for the current density in the dark and under light illumination, respectively. The V_{eff} was obtained from the equation $V_{eff} = V_0 - V_{appl}$, where V_0 is the voltage at which J_{ph} is 0, and V_{appl} represents the applied voltage. P_{diss} was calculated using the equation $P_{diss} = J_{ph}/J_{sat}$, where J_{ph} is the photocurrent under short-circuit conditions and J_{sat} is the saturated photocurrent in the high V_{eff} region.

Light Intensity Dependence of V_{oc} and J_{sc}

The degree of trap-assisted recombination and bimolecular recombination was also investigated by light intensity dependence of V_{oc} and J_{sc}.[7,8] The trap-assisted recombination can be described by the relationship between V_{oc} and P_{light} in a formula of $V_{oc} \propto nkT/q\ln(P_{light})$, where k, q, and T are the Boltzmann constant, elementary charge, and Kelvin temperature, respectively. If n value is equal to 1, the trap-assisted recombination is negligible. If n value is equal to 2, the trap-assisted recombination is dominant. The bimolecular recombination can be described by the relationship between J_{sc} and P_{light} in a formula of $J_{sc} \propto (P_{light})^{\alpha}$. If an α value is 1, the charge carriers can be collected efficiently at the electrodes by avoiding bimolecular recombination (bimolecular recombination is minimum).

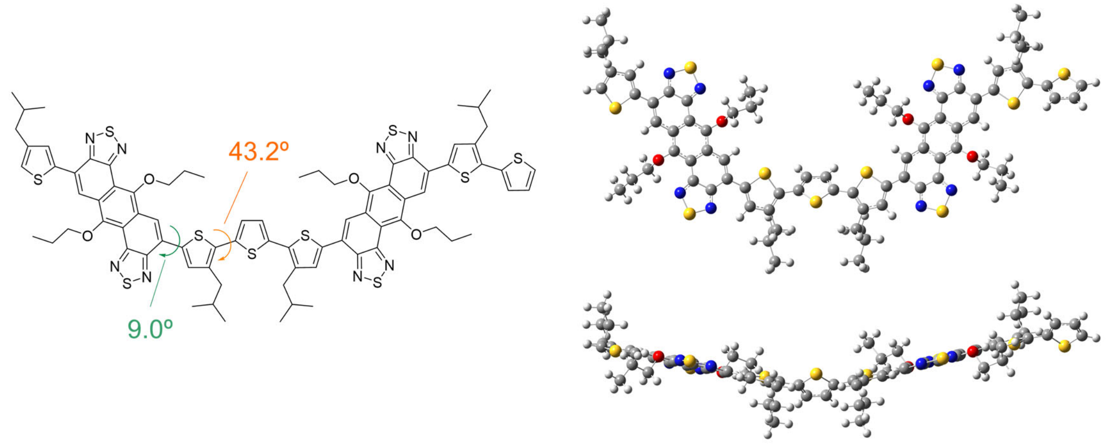

Figure S5. Optimized molecular geometries of the model compound of **PATz3T**, calculated using DFT at B3LYP/6-311G(d).

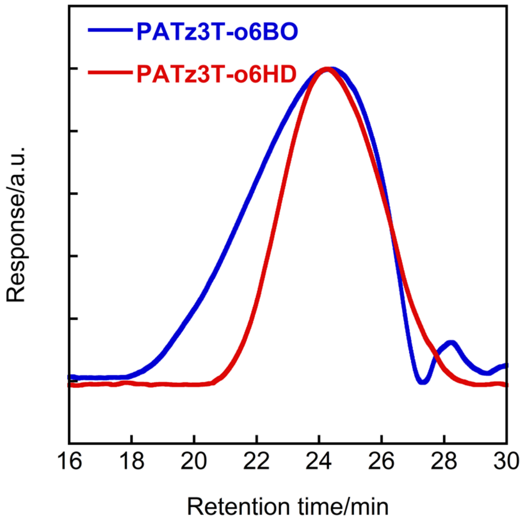

Figure S6. GPC curves of **PATz3T-o6BO** and **PATz3T-o6HD**.

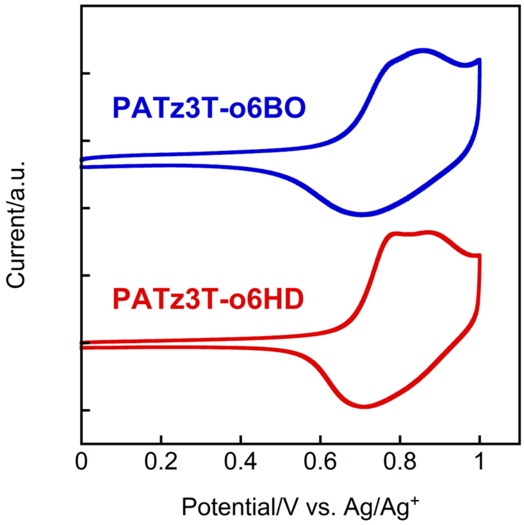

Figure S7. Cyclic voltammograms of **PATz3T-o6BO** and **PATz3T-o6HD** in thin film.

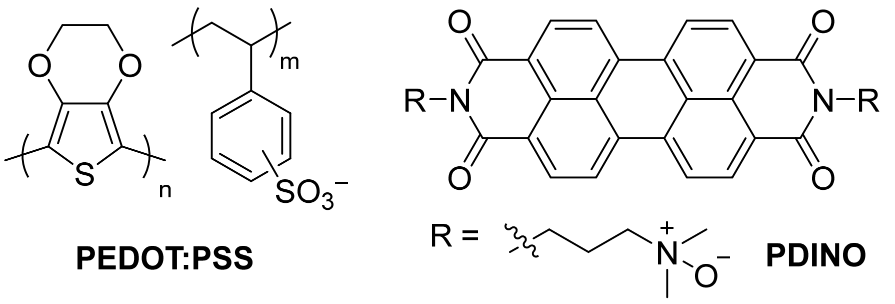

Figure S8. Chemical structures of **PEDOT:PSS** and **PDINO**.

Table S1. Solar cell performances of **PATz3T-o6BO**:**Y12**-based devices with the configuration of ITO/(PEDOT:PSS)/**PATz3T-o6BO**:**Y12**/**PDINO**/Ag

p/n[a]	solvent (v/v)[b]	TA	J_{sc}/mA cm^{-2}	V_{oc}/V	FF	PCE_{max} (PCE_{avg})/%
1:1.2	CF	-	9.82	0.87	0.37	3.18 (3.08)
1:1.2	CF+0.25% DIO	-	12.55	0.86	0.38	4.10 (3.92)
1:1.2	CF+0.25% DIO	[c]	14.01	0.86	0.38	4.60 (4.42)
1:1.2	CF+0.5% DIO	-	12.94	0.84	0.39	4.23 (3.94)
1:1.2	CB+0.5% DIO	[c]	12.96	0.81	0.36	3.76 (3.65)
1:1.5	CB+0.5% DIO	[c]	15.62	0.83	0.40	5.21 (5.02)
1:2	CB+0.5% DIO	[c]	16.21	0.82	0.40	5.34 (5.29)

[a]Weight ratios of **PATz3T-o6BO** and **Y12**. [b]CF = chloroform. DIO = 1,8-diiodooctane. [c]Thermal annealing at 100 °C for 10 min.

Table S2. Solar cell performances of **PATz3T-o6HD**:**Y12**-based devices with the configuration of ITO/(PEDOT:PSS)/**PATz3T-o6HD**:**Y12**/**PDINO**/Ag

p/n[a]	solvent (v/v)[b]	TA	J_{sc}/mA cm^{-2}	V_{oc}/V	FF	PCE_{max} (PCE_{avg})/%
1:1.2	CF	-	10.68	0.88	0.37	3.48 (3.30)
1:1.2	CF+0.25% DIO	-	13.19	0.86	0.42	4.77 (4.62)
1:1.2	CF+0.25% DIO	[c]	14.57	0.86	0.43	5.36 (5.20)
1:1.2	CF+0.5% DIO	-	16.81	0.85	0.47	6.77 (6.51)
1:1	CF+0.5% DIO	-	15.41	0.85	0.44	5.85 (5.63)
1:1.5	CF+0.5% DIO	-	16.88	0.85	0.45	6.48 (6.27)
1:2	CF+0.5% DIO	-	18.77	0.84	0.45	7.05 (6.76)
1:2	CF+0.5% DIO	[c]	18.45	0.83	0.47	7.20 (6.93)
1:2	CF+0.75% DIO	-	18.60	0.83	0.47	7.34 (7.18)
1:2	CF+0.75% DIO	[c]	19.28	0.82	0.49	7.69 (7.44)
1:2	CF+1% DIO	-	17.79	0.83	0.50	7.33 (7.16)
1:2	CF+1% DIO	[c]	5.19	0.59	0.30	0.92 (0.56)
1:2	CB+0.75% DIO	[c]	19.51	0.83	0.49	7.94 (7.51)
1:1.2	CB+0.5% DIO	[c]	16.89	0.86	0.47	6.81 (6.60)
1:1.5	CB+0.5% DIO	[c]	18.00	0.84	0.47	7.02 (6.91)
1:2	CB+0.5% DIO	[c]	20.04	0.83	0.47	7.83 (7.56)

[a]Weight ratios of **PATz3T-o6HD** and **Y12**. [b]CF = chloroform. DIO = 1,8-diiodooctane. [c]Thermal annealing at 100 °C for 10 min.

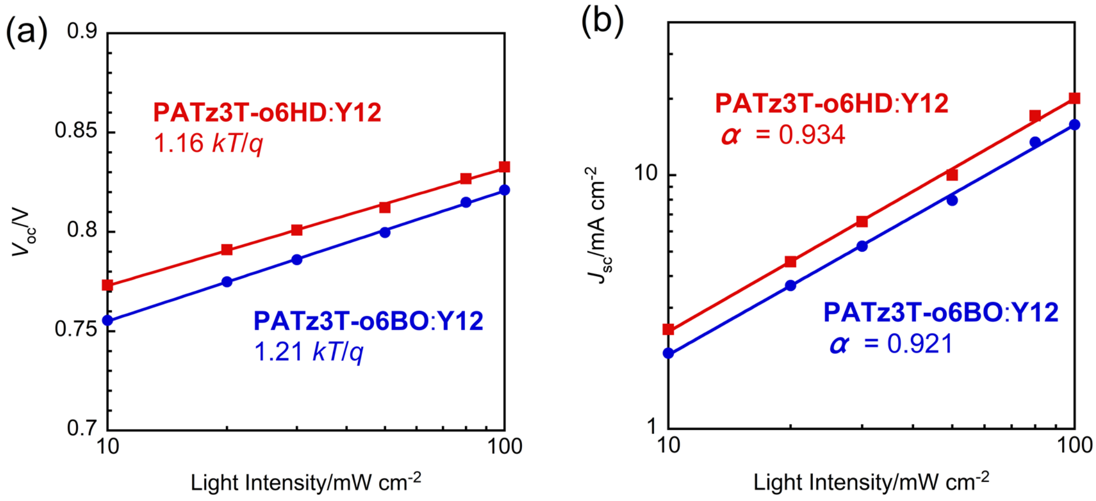

Figure S9. Light intensity dependence of (a) V_{oc} and (b) J_{sc} in **PATz3T**:**Y12**-based OPVs.

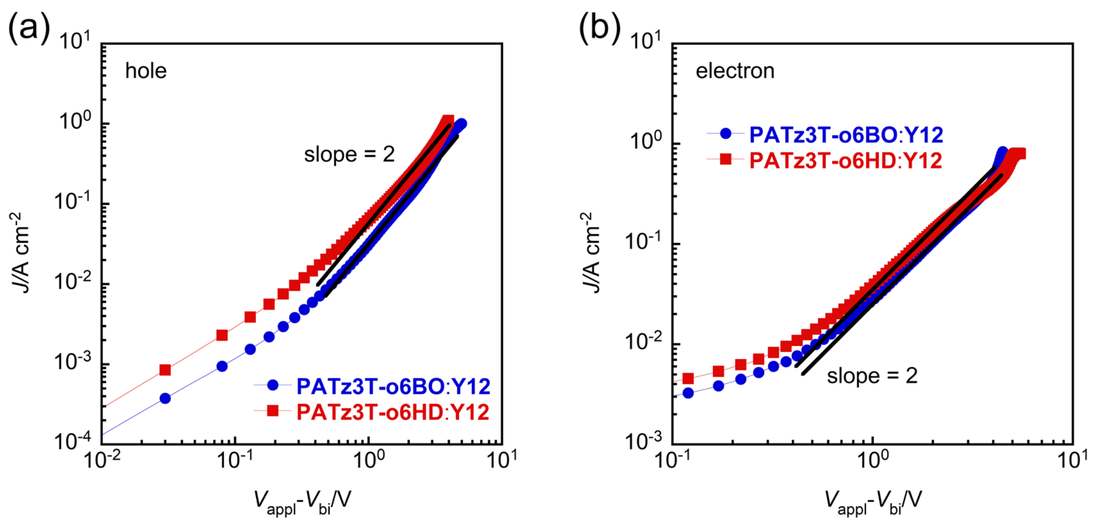

Figure S10. *J*-*V* characteristics of **PATz3T-o6BO** and **PATz3T-o6HD**-based hole- and electron-only devices with configurations of ITO/(PEDOT:PSS)/(**PATz3T-o6BO** or **PATz3T-o6HD**:**Y12**)/MoO_3/Ag and ITO/ZnO/(**PATz3T-o6BO** or **PATz3T-o6HD**:**Y12**)/**PDINO**/Ag; (a) hole-only and (b) electron-only devices.

References

1. H. Mori, R. Hosogi, Y. Minagawa, H. Yamane, Y. Nishihara, *ACS Appl. Polym. Mater.* **2024**, *6*, 3883.
2. J. Qi, X. Zhou, D. Yang, W. Qiao, D. Ma, Z.-Y. Wang, *Adv. Funct. Mater*. **2014**, *24*, 7605.
3. M. J. Frisch, G. W. Trucks, H. B. Schlegel, G. E. Scuseria, M. A. Robb, J. R. Cheeseman, G. Scalmani, V. Barone, B. Mennucci, G. A. Petersson, H. Nakatsuji, M. Caricato, X. Li, H. P. Hratchian, A. F. Izmaylov, J. Bloino, G. Zheng, J. L. Sonnenberg, M. Hada, M. Ehara, K. Toyota, R. Fukuda, J. Hasegawa, M. Ishida, T. Nakajima, Y. Honda, O. Kitao, H. Nakai, T. Vreven, J. A. Montgomery, J. E. Peralta, F. Ogliaro, M. Bearpark, J. J. Heyd, E. Brothers, K. N. Kudin, V. N. Staroverov, T. Keith, R. Kobayashi, J. Normand, K. Raghavachari, A. Rendell, J. C. Burant, S. S. Iyengar, J. Tomasi, M. Cossi, N. Rega, N. J. Millam, M. Klene, J. E. Knox, J. B. Cross, V. Bakken, C. Adamo, J. Jaramillo, R. Gomperts, R. E. Stratmann, O. Yazyev, A. J. Austin, R. Cammi, C. Pomelli, J. W. Ochterski, R. L. Martin, K. Morokuma, V. G. Zakrzewski, G. A. Voth, P. Salvador, J. J. Dannenberg, S. Dapprich, A. D. Daniels, O. Farkas, J. B. Foresman, J. V. Ortiz, J. Cioslowski, D. J. Fox, Gaussian 09, Revision D. 01, Gaussian, Inc., Wallingford, CT, **2013**.
4. X. Song, Y. Song, H. Xu, S. Gao, Y. Wang, J. Li, J. Hai, W. Liu, W. Zhu, *Adv. Energy Mater.* **2023**, *13*, 2203009.
5. Y. Sun, J. H. Seo, C. J. Takacs, J. Seifter, A. J. Heeger, *Adv. Mater.* **2011**, *23*, 1679.
6. H. Lu, H. Wang, G. Ran, S. Li, J. Zhang, Y. Liu, W. Zhang, X. Xu, Z. Bo, *Adv. Funct. Mater.* **2022**, *32*, 2203193.
7. S. R. Cowan, A. Roy, A. J. Heeger, *Phys. Rev. B* **2010**, *82*, 245207.
8. L. J. A. Koster, V. D. Mihailetchi, R. Ramaker, P. W. Blom, *Appl. Phys. Lett*. **2005**, *86*, 123509.
